# Primary School-Aged Children’s Physical Activity Level, Eating Habit, and Sleeping Pattern Changes During the COVID-19 Pandemic in Indonesia

**DOI:** 10.7759/cureus.53354

**Published:** 2024-01-31

**Authors:** Rini Sekartini, Clarissa J Aditya, Billy Pramatirta, Stephanie Gosal, Kevin Tjoa

**Affiliations:** 1 Department of Pediatrics, Faculty of Medicine, Cipto Mangunkusumo Hospital, Universitas Indonesia, Jakarta, IDN; 2 Faculty of Medicine, Universitas Indonesia, Jakarta, IDN

**Keywords:** children, sleeping patterns, eating habits, physical activity, pandemic, covid-19

## Abstract

Background and objective

This study aims to explore the effect of physical distancing on physical activity, eating habits, and sleeping patterns among Indonesian primary schoolchildren during the COVID-19 pandemic.

Methodology

This cross-sectional study was conducted from October to December 2020, involving 489 primary schoolchildren. Parents/caregivers were queried about changes in their children's physical activity (utilizing the Physical Activity Questionnaire for Older Children - PAQ-C), eating habits (via a questionnaire modified from Southeast Asian Nutrition Surveys - SEANUTS), and sleeping patterns (assessed using the Children's Sleep Habits Questionnaire - CSHQ) both before and during the pandemic. Various sociodemographic characteristics and income status were also obtained. Paired univariate and multivariate analyses were conducted where applicable.

Results

In comparison to the period before the pandemic, both the PAQ-C score and active proportion significantly decreased during the pandemic (*P *= 0.000). Children consumed more snacks (322, 65.8%) but less canned and processed foods (180, 36.8%, and 128, 26.2%, respectively). Sleep duration and sufficiency increased significantly, with a CSHQ score mean of 48.62 ± 9.88 (*P *= 0.000, *P *= 0.004), and sleep disturbance was observed in 391 (79.96%) subjects.

Conclusions

Children were physically less active during the COVID-19 pandemic. They also experienced variable changes in eating habits based on parental employment, family income and expenses, and the presence of governmental support. While more children had longer sleep duration and more achieved the recommended sleep time, sleep disturbance happened in most subjects.

## Introduction

The COVID-19 pandemic, which started in March 2020, extended until May 2023, and it has now entered the endemic phase. Preventive measures and regulations have been enforced in every country, including Indonesia. Since then, Indonesia has been implementing a large-scale social restriction (*Pembatasan Sosial Berskala Besar* [PSBB]) program, which includes a study/work-at-home policy and public place restriction [1]. Several studies have found changes in children’s lifestyle during school holidays, for instance, increased body mass index (BMI) [2], prolonged screen time, decreased vegetable consumption, and increased sugar consumption [3]. It was presumed that school may offer a structured environment for practicing physical activity, eating habits, and sleeping patterns, which are strongly associated with the risk of obesity [4].

The World Health Organization (WHO) recommends that children engage in moderate to vigorous physical activity (MVPA) for 60 minutes every day [5]. It is known that physical activity is needed to ensure children’s immunity, mental health, and growth and development [6]. In addition, a balanced and healthy diet could reduce the risk of obesity in children, as well as non-communicable diseases, such as hypertension, cardiovascular diseases, cancer, and others [7]. Furthermore, a disruption in circadian rhythm due to an unstructured sleeping pattern could increase the risk of obesity in children [8]. As habitual health practices are formed and developed in childhood, it is necessary to promote adequate physical activity, healthy eating habits, and regular sleeping patterns for children [9].

In Indonesia itself, there is limited data on the effect of physical distancing due to COVID-19 on physical activity, eating habits, and sleeping patterns in preliminary schoolchildren. Meanwhile, studies have shown the possibility of changes in children’s lifestyle during study-at-home policy due to physical distancing regulation. Hence, this study aimed to explore the effect of physical distancing on physical activity, eating habits, and sleeping patterns among primary schoolchildren in Indonesia during the COVID-19 pandemic, using subjective measures for both pre-pandemic and during the pandemic periods.

## Materials and methods

Data collection

A cross-sectional study was performed by using online questionnaires from October to December 2020, aimed at parents with children aged 6-12 years in Indonesia. The online questionnaire was designed in the Indonesian language using the Google Form service, with a multi-page setting that restricted the respondents to moving to the next page if they failed to fit into the requirements of the current page. The informed consent form was provided on the front page, and if respondents agreed to give consent, they could proceed to the next page. Consecutive sampling was used in this study, as every eligible respondent was included in this study until the desired sample size was obtained. The minimum sample size was calculated as 432 with Za = 1.96, Zb = 0.84, and P1 = 0.5 (because no previous studies were available). Respondents were included in this study if they had children aged 6-12 years, and the children were enrolled in primary school during the pandemic. The responses were excluded if they did not provide consent to participate in the study. This study protocol was approved by the Ethical Committee of the Faculty of Medicine, Universitas Indonesia.

This study collected demographic data on the child’s gender, birthdate, current level of education (grade in primary school), and parental employment status (working at home/office/unemployed), both before and during the pandemic. Data on parental income and governmental support both before and during the pandemic were also obtained. Children’s physical activity level was measured with the Physical Activity Questionnaire for Older Children (PAQ-C) questionnaire, a self-reported seven-day recall of nine items, each scored from 1 to 5, following the scoring rules of Kowalski et al. [10]. Data on parental perception of their children’s physical activity level and screen time were also collected. Meanwhile, children’s eating habits were measured by using the eating habit survey section from the Southeast Asian Nutrition Survey (SEANUTS) [11] study with permission from the investigators. Next, the sleeping pattern was collected with self-reported sleeping duration and measured with the Children's Sleep Habits Questionnaire (CSHQ) by Owens et al. [12]. A total score of CSHQ was obtained, and a cutoff score of 41 was used to determine the presence of sleeping disturbances. In the end, a total of 489 eligible respondents were included in this study for data analysis.

Data analysis

Descriptive statistics were reported in percentages, and in cases where the distribution was not normal, median values with 25th and 75th percentiles were reported instead of mean and standard deviation (SD). Univariate analysis was carried out using McNemar for dichotomous paired dependent variables, chi-square analysis was preferred for nominal or categorical variables, and paired t-test was chosen for numeric paired dependent variables. Variables with *P*-value ≤ 0.25 in univariate analysis were included for further multivariate analysis, which was carried out using binomial logistic regression. When available, effect estimates were presented in adjusted odds ratio (OR) and confidence interval (CI) of 95%. All statistical analyses were conducted using IBM SPSS Statistics for Windows, Version 24.0 (IBM Corp., Armonk, NY), and a *P*-value of 0.05 was considered significant. We analyzed sex, class, gadget exposure, parental employment, government support, and family income in association with physical activity, eating habits, and sleeping duration and disturbance.

## Results

Subject demographics

A total of 489 children, evenly distributed between females (243, 49.7%) and males (246, 50.3%), spanning from first to sixth grade in primary school, were analyzed. Most of them were second (121, 24.74%) and sixth (103, 21.06%) graders. It was reported that most children had one of their parents work/stay at home during the pandemic (247, 50.5%). There were a significant number of parents (father and mother) who shifted from working outside before the pandemic to working from home during the pandemic (McNemar test, *P*-value < 0.001), while only 73 (14.93%) families received governmental support during the later situation. Up to 416 (85.1%) parents said that their children were more exposed to gadgets, while only 7 (1.4%) children had less screen time. Detailed subject demographics are found in Table 1.

**Table 1:** Subjects' demographics. N/A not applicable

No.	Characteristics		Measurement	*P*-value (OR, 95% CI)	Notes
1	Sex (*n*, %)	Male	246 (50.3)	N/A	-
		Female	243 (49.7)		
2	Grade (*n*, %)	1	60 (12.27)	N/A	-
		2	121 (24.74)		
		3	69 (14.11)		
		4	74 (15.13)		
		5	62 (12.68)		
		6	103 (21.06)		
3	Age (mean, SD)		8.81 (1.74)	N/A	-
4	Parental employment during the pandemic (*n*, %)	Both work outside home	91 (18.6)	N/A	-
		One stay/work at home	247 (50.5)		
		Both stay/work at home	151 (30.9)		
5	Father's employment (*n*, %)	Pre-pandemic (out/in)	411 (84.0)/78 (16.0)	<0.001	McNemar
		During the pandemic (out/in)	283 (57.9)/206 (42.1)		
6	Mother's employment (*n*, %)	Pre-pandemic	240 (49.1)/249 (50.9)	<0.001 (185.25; 45.0-762.8)	McNemar
		During the pandemic	146 (29.9)/343 (70.1)		
7	Family income during the pandemic	Decreased	247 (50.5)	N/A	-
		Unchanged	225 (46)		
		Increased	17 (3.5)		
8	Family expense during the pandemic	Decreased	68 (13.9)	N/A	-
		Unchanged	159 (32.5)		
		Increased	262 (53.6)		
9	Governmental support (*n*, %)	Yes	73 (14.93)	N/A	-
10	Gadget exposure (*n*, %)	Decreased	7 (1.4)	N/A	-
		Unchanged	66 (13.5)		
		Increased	416 (85.1)		

Comparing before and during the pandemic, subjects’ behaviors changed as follows: (1) PAQ-C score and active proportion decreased significantly during the pandemic; (2) for the majority of subjects, almost all types of food consumption were not changed during the pandemic, with exception of snacks, which were found to increase in 322 (65.8%) subjects, while consumption of canned and instant food decreased the most (180, 36.8%, and 128, 26.2%, respectively); and (3) the sleep duration and sufficiency increased significantly with CSHQ score means of 48.62 ± 9.88 and presence of sleep disturbance was observed in 391 (79.96%) subjects. Further details of subjects’ physical activity, food consumption, and sleep patterns are portrayed in Table 2.

**Table 2:** Subjects’ behaviors: physical activity, food consumption, and sleep pattern pre-pandemic and during the pandemic. N/A, not applicable; OR, odds ratio; CI, confidence interval; PAQ-C, Physical Activity Questionnaire for Older Children; CSHQ, Children's Sleep Habits Questionnaire

No.	Behaviors’ parameter	Pre-pandemic/during the pandemic	Measurement	*P*-value (OR; 95% CI)	Notes
Physical activity					
1	PAQ-C (mean, SD)	Pre-pandemic	2.72 (0.71)	<0.001	Paired t-test
		During the pandemic	2.47 (0.75)		
2	PAQ-C (Active/inactive) (*n*, %)	Pre-pandemic	405 (82.82)/84 (17.18)	<0.001 (28.225; 14.550-54.754)	McNemar
		During the pandemic	346 (70.76)/143 (29.24)		
Food consumption (decreased/unchanged/increased) (*n*, %)					
1	Vegetables	During the pandemic	34 (7)/333 (68.1)/122 (24.9)	N/A	N/A
2	Fruits	During the pandemic	30 (6.1)/249 (50.9)/210 (42.9)		
3	Meat and seafood	During the pandemic	22 (4.5)/304 (62.2)/163 (33.3)		
4	Egg	During the pandemic	9 (1.8)/254 (51.9)/226 (46.2)		
5	Dairy products	During the pandemic	28 (5.7)/235 (48.1)/226 (46.2)		
6	Canned food	During the pandemic	180 (36.8)/263 (53.8)/46 (9.4)		
7	Instant food	During the pandemic	128 (26.2)/258 (52.8)/103 (21.2)		
8	High-sugar drinks	During the pandemic	97 (10.8)/276 (56.4)/116 (23.7)		
9	Snacks	During the pandemic	29 (5.9)/138 (28.2)/322 (65.8)		
Sleep pattern					
1	Sleep duration (mean, SD)	Pre-pandemic	9.06 (1.68)	<0.001	Paired t-test
		During the pandemic	9.31 (1.48)		
2	Sleep duration (enough/not enough) (*n*, %)	Pre-pandemic	273 (55.83)/216 (44.17)	0.004 (8.174; 5.38-12.42)	McNemar
		During the pandemic	306 (62.58)/183 (37.42)		
3	CSHQ score (mean, SD)	During the pandemic	48.62 (9.88)	N/A	N/A
4	Sleep disturbance presence (CSHQ) (*n*, %)	During the pandemic	391 (79.96)	N/A	N/A

Physical activity

For physical activity, a statistically significant correlation was found between the COVID-19 pandemic and changes in PAQ-C score (*P *< 0.001). It appeared that 334 out of 489 respondents (68.30%) remained physically active, whereas 71 out of 489 respondents (14.52%) had become physically inactive since the pandemic. The odds of becoming physically inactive since the pandemic began increased up to 28 times (OR 28.225, 95% CI 14.550-54.754).

Another statistically significant correlation was also found between parental perception of children’s physical activity and changes in the PAQ-C score (*P *< 0.001). It appeared that most parents perceived their children to be physically inactive since the pandemic (330/489 respondents), with 71.5% of them having an accurate perception of their children's current physical activity level. The odds of parental perception of their children being physically inactive since the pandemic increased up to two times (OR 2.607, 95% CI 1.761-3.859). Other details are shown in Table 3.

**Table 3:** Physical activity status (based on PAQ-C) among different demographic groups during the pandemic. N/A, not applicable; OR, odds ratio; CI, confidence interval; PAQ-C, Physical Activity Questionnaire for Older Children

No.	Categories		Physical activity (PAQ-C)					
			Descriptive		Univariate		Multivariate	
			Decreased	Unchanged/increased	*P*-value	OR (95% CI)	*P*-value	OR (95% CI)
1	Sex (*n*, %)	Male (ref)	151 (61.4)	95 (38.6)	0.189	1.282 (0.885-1.857)	0.573	1.120 (0.755-1.662)
		Female	163 (67.1)	80 (32.9)				
2	Grade (*n*, %)	Grade 1 (ref)	34 (56.7)	26 (43.3)	0.101	N/A	N/A	
		Grade 2	72 (59.5)	49 (40.5)			0.872	1.056 (0.546-2.041)
		Grade 3	54 (78.3)	15 (21.7)			0.006	3.106 (1.383-6.976)
		Grade 4	45 (60.8)	29 (39.2)			0.824	1.086 (0.523-2.255)
		Grade 5	41 (66.1)	21 (33.9)			0.371	1.420 (0.658-3.063)
		Grade 6	68 (66.0)	35 (34.0)			0.300	1.441 (0.722-2.878)
3	Parental employment (*n*, %)	Both work outside home (ref)	61 (67.0)	30 (33.0)	0.562	N/A	N/A	
		One stay/work at home	151 (61.1)	96 (38.9)			0.356	0.776 (0.453-1.330)
		Both stay/work at home	102 (67.5)	49 (32.5)			0.661	1.140 (0.634-2.049)
4	Parental perception on children's physical activity (*n*, %)	Decreased	236 (71.5)	94 (28.5)	0.00	2.61 (1.761-3.859)	0.000	2.807 (1.818-4.334)
		Unchanged/Increased (ref)	78 (49.1)	81 (50.9)				
5	Family income during the pandemic (*n*, %)	Decreased	167 (67.6)	80 (32.4)	0.113	1.349 (0.931-1.955)	0.049	1.519 (1.001-2.305)
		Unchanged/increase (ref)	147 (60.7)	95 (39.3)				
6	Family expense during the pandemic (*n*, %)	Decreased	38 (55.9)	30 (44.1)	0.122	0.665 (0.396-1.118)	0.047	0.555 (0.311-0.993)
		Unchanged /increased (ref)	276 (65.6)	145 (34.4)				
7	Governmental support (*n*, %)	Yes (ref)	51 (69.9)	22 (30.1)	0.275	1.349 (0.787-2.31)	0.118	1.593 (0.888-2.857)
		No	263 (63.2)	153 (36.8)				
8	Gadget exposure (*n*, %)	Unchanged/decreased (ref)	41 (56.2)	32 (43.8)	0.12	1.49 (0.90-2.468)	0.976	0.991 (0.559-1.759)
		Increased	273 (65.6)	143 (34.4)				

Similar findings were found after multivariate analysis on physical inactivity (adjusted for sex, grade, parental employment, parental perception of children’s physical activity, family income and expense, governmental support, and gadget exposure), where parental perception of physical inactivity on their children, as one of the independent factors, increased the risk of inactivity during pandemic up to two times. Similarly, sex, grade, parental employment, family income and expense, governmental support, and gadget exposure were also shown as independent factors of physical inactivity. Alternatively, decreased family expense during the pandemic was a protective factor against physical inactivity (OR 0.555, 95% CI 0.311-0.993), whereas decreased family income during the pandemic increased 1.5 times the risk of physical inactivity. Third graders exhibited a higher risk of physical inactivity compared to other independent factors (OR 3.106, 95% CI 1.383-6.976).

Eating habits

Some analysis of eating habits showed that egg consumption in girls increased compared to boys (*P *< 0.05). Different grades were also found to be a significant factor affecting vegetable consumption (*P *< 0.05). Moreover, parental employment affected vegetable consumption (*P *< 0.05). Changes in family income and expenses after the pandemic affected some eating habits. Family income significantly affected cereal consumption (*P *< 0.05). Meanwhile, family expenses affected fruits, meat and seafood, egg, dairy products, canned food, instant food, and sweet drinks consumption plus snack portions (*P *< 0.05). The details of the statistical significance are given in Table 4.

**Table 4:** P-values for eating habits and food consumption among different demographic groups during the pandemic. ^*^P < 0.05. ^**^P < 0.25.

Variable	Vegetables	Fruits	Egg	Meat and seafood	Cereal	Dairy products	Canned food	Instant food	Sweet drinks	Meal portion	Snack portion
Sex	0.734	0.302	0.008*	0.25**	0.389	0.081**	0.965	0.687	0.177**	0.295	0.341
Grade	0.01*	0.685	0.87	0.919	0.478	0.053**	0.499	0.834	0.967	0.61	0.478
Parental employment	0.045*	0.138	0.396	0.771	0.822	0.2**	0.238**	0.863	0.111**	0.672	0.594
Governmental support	0.516	0.233**	0.111**	0.929	0.869	0.658	0.026**	0.15**	0.162**	0.597	0.104**
Family income	0.16**	0.156**	0.064**	0.474	0.01*	0.287	0.084**	0.952	0.064**	0.531	0.623
Family expense	0.476	0.006*	0.001*	0.001*	0.274	0.038*	0.001*	0.001*	0.003*	0.218	0.001*

Further multivariate analyses were conducted on variables with *P* < 0.25 on univariate analysis. Fourth graders(OR 3.63, 95% CI 1.55-8.50) and having both parents working/staying at home (OR 3.13, 95% CI 1.58-6.21) were found to increase the odds of eating vegetables. Increased family expense during the pandemic (OR 1.88, 95% CI 1.24-2.84) was associated with increased odds of eating fruits. Girls were found to eat more eggs than boys (OR 1.59, 95% CI 1.10-2.32); decreased (OR 1.95, 95% CI 1.05-3.62) and increased (OR 2.71, 95% CI 1.77-4.14) expenses were both associated with an increase in egg consumption. Increased family expenses were also associated with increased meat and seafood consumption (OR 2.76, 95% CI 1.77-4.32) and dairy product consumption (OR 1.62, 95% CI 1.07-2.44). Third graders were also found to have an increased dairy product consumption (OR 2.47, 95% CI 1.21-5.04) compared to others. Decreased (OR 6.33, 95% CI 2.01-19.95 and OR 7.30, 95% CI 2.41-22.09) and increased (OR 3.54, 95% CI 1.32-9.51, and OR 3.78, 95% CI 1.42-10.05) family expenses were found to increase canned food and instant food consumption, respectively. Children whose families did not receive any governmental support were found to have a decreased consumption of canned foods (OR 0.29, 95% CI 0.11-0.77) and instant foods (OR 0.27, 95% CI 0.10-0.69) compared to those who received financial support. Sweet drink consumption was increased in children whose families had increased expenses (OR 2.35, 95% CI 1.39-3.99) and whose one of the parents stayed at home (OR 1.98, 95% CI 1.05-3.74). 

Sleeping patterns

During the pandemic, significant improvements in sleep duration among respondents were noted. Compared to pre-pandemic, children had longer sleeping time ( 9.31 ± 1.48 hours, mean ± SD) and a significantly higher number of children had enough sleep (9-12 hours) during the pandemic (55.83% to 62.58%, *P *= 0.004, McNemar test). The odds of having enough sleep during the pandemic increase up to eight times (OR 8.174, 95% CI 5.38-12.42). Excessive sleep was relatively maintained (14 vs 13, pre vs during the pandemic). What we found interesting is, that while sleeping duration was improved, the CSHQ score interpretation suggests that most children (391, 79.96%) had some degree of sleep disturbances (mean CSHQ score of 48.62).

No significant association was found between sex and sleep duration, nor was there a significant association with the presence of sleep disturbances. On the other hand, the association between sleep duration and sleep disturbance with students’ class (grader) was significant in univariate analysis (*P *= 0.009 and 0.024, respectively). Although no linearity was observed, there was a trend that higher graders tend to have less adequate sleep duration. No similar pattern was found in sleep disturbance. Our analysis showed that sleep inadequacy was correlated with the presence of sleep disturbance (OR 1.82, 95% CI 1.16-2.83; data not shown). Based on the univariate analysis, children with increased gadget exposure were twice (OR 2.01, 95% CI 1.14-3.54) more likely to experience inadequate sleep duration and 1.5 times more likely to have sleep disturbance as implied by the CSHQ score although the latter association was not significant (*P *= 0.166). The details are given in Table 5.

**Table 5:** Sleeping patterns in different demographic groups during the pandemic; analytical results. N/A, not applicable; OR, odds ratio; CI, confidence interval; CSHQ, Children's Sleep Habits Questionnaire

No.	Variables		Sleep duration				CSHQ interpretation			
			Univariate		Multivariate		Univariate		Multivariate	
			*P*-value	OR (95% CI)	*P*-value	OR (95% CI)	*P*-value	OR (95% CI)	*P*-value	OR (95% CI)
1	Sex (*n*, %)	Male (ref)	0.991	1.002 (0.695-1.446)	N/A		0.061	0.653 (0.417-1.021)	0.796	1.051 (0.721-1.532)
		Female								
2	Grade (*n*, %)	1 (ref)	0.009	N/A	N/A		0.024	N/A	N/A	
		2			0.007	0.383 (0.191-0.767)			0.02	2.827 (1.177-6.786)
		3			0.104	0.637 (0.37-1.097)			0.002	3.083 (1.531-6.208)
		4			0.039	0.512 (0.271-0.967)			0.032	2.347 (1.074-5.131)
		5			0.004	0.385 (0.202-0.734)			0.254	1.505 (0.746-3.035)
		6			0.393	0.758 (0.402-1.431)			0.751	1.123 (0.548-2.304)
3	Gadget exposure (*n*, %)	Unchanged/decreased (ref)	0.015	2.009 (1.139-3.542)	0.049	0.558 (0.312-0.998)	0.166	1.5 (0.843-2.647)	0.023	0.492 (0.266-0.908)
		Increased								
4	Parental employment (*n*, %)	Both work outside home (ref)	0.684	N/A	N/A		0.569	N/A	N/A	
		One stay/work at home								
		Both stay/work at home								

Binomial logistic regression on sleep duration adequacy (adjusted for grade, family income, and gadget exposure) showed that compared to the first grader, sixth graders had a risk of 2.6 times to have sleep inadequacy (*P *= 0.006) and gadget exposure still correlated significantly with lower sleep duration (OR 1.8, 95% CI 1.006-3.229). Multivariate analysis on sleep disturbance (adjusted for sex, grade, family income, expense, governmental support, and gadget exposure) showed sex, grade, and gadget exposure as independent factors. Being female was a protective factor to sleep disturbance, while higher gadget exposure increased up to two times the risk of sleep disturbance. However, it is important to note that being in the sixth grade was a protective factor against sleep disturbance (OR 0.353, 95% CI 0.146-0.855) despite a risk factor for sleep duration inadequacy. Parental employment, representing parents' availability at home, family income and expense, and governmental support, was not significantly associated with either sleep duration or sleep disturbance.

## Discussion

Physical activity

During the early period of the COVID-19 pandemic, a significant reduction in physical activity was found among children [13]. This reduction varied significantly according to the group of age, where preschool children experienced the slightest change [13]. Nonetheless, this variety has also been found in studies in other countries [14].

In our study, the majority of the respondents did not have a change in their physical activity level during the pandemic, and most of them remained physically active. The plausible explanation was the limited adherence to the self-protection measures throughout Indonesia [15]. Furthermore, a study in Croatia [16] showed that previously active children were more likely to remain physically active during the pandemic.

A similar finding in Canada suggested a decrease in children’s physical activity happened in the early period of the pandemic and would increase in the latter period [17]. Children who experienced an increase in physical activity during the pandemic in our study were more likely influenced by parental support. Similarly, children in Canada [17] also reported an increase in physical activity, whether outdoor or indoor, during the pandemic. The study-at-home regulation brought children to spend more time together with their families, especially their parents [14]. Parents had a major role in supporting children’s physical activity through their financial resources and influencing their children by encouraging and co-participating in children’s physical activity [14].

Several children in our study had also experienced a decrease in physical activity during the pandemic. This result was consistent with other studies, such as in Poland [18] and Switzerland [19]. This may happen due to the social restriction and the respondent’s adherence to COVID-19 preventive measures [16]. Social restriction means limiting access to children’s playmates, including school closures [20]. Moreover, considering the family's influential role in children’s physical activity, the negative association of parental employment and family conflict might also contribute to a decrease in physical activity [14,20]. It should be noted, that even in the mid-period of the COVID-19 pandemic, children are still experiencing a decrease in physical activity. Hence, the pandemic might have a lasting impact on children’s physical activity and might lead to an unhealthy new normal.

Eating habits

After post hoc analysis, it was found that children whose family’s expenses during the pandemic increased had an increase in instant food and meat and seafood consumption. However, our findings were contrary to a study in Poland where strict social restrictions resulted in reduced vegetable and fruit consumption and access to fast-food restaurants [18]. Snack portions also increased during the pandemic in our study, similar to a study in Switzerland [19]. These results suggested that more analysis may be needed to seek the reason behind the differences between countries' pandemic regulation and implementation and the subsequent children's eating habits.

Sleeping patterns

Sleep Duration and CSHQ

There are two important components in sleeping: quantity and quality. The American Academy of Sleep Medicine recommends a sleeping duration of 9-12 hours for children aged 6-12 years [9]. We found a significant positive association between sleep duration and sleep disturbance based on the CSHQ score (higher sleep duration associated with higher sleep disturbance), which deviates from the theory. This finding indicates that although the quantity aspect is fulfilled, the quality may be impaired. Sleeping quality is a different parameter, but also an integral aspect of sleep. Moreover, since CHSQ has a lower sensitivity compared to polysomnography or actigraphy to detect sleep disturbance, the actual sleep disturbance rate may be higher [21]. In addition, sleep duration and quality are not the only important aspects of sleep. Sleep consistency is also a vital factor, especially for academic performance, which was not measured in our study [22].

Pandemic Effect on Sleep Duration and Disturbance

The pandemic, leading to online learning activities for children, might alter the daily routine and impact sleeping patterns, specifically sleeping timing [23-24]. Our study suggests that the pandemic has improved sleep duration, as more children now achieve sufficient sleep duration. This finding was similar to what Kaditis et al. [23] observed: increased sleep duration during the pandemic. Unfortunately, the majority of children in our study (391, 79.96%) had sleep disturbance during the pandemic. This was higher than a systematic review that reported a prevalence of 54% [25]. While we have no data before the pandemic, previous studies found different trends in sleep disturbance. Di Giorgio et al. [24] suggested no change in sleep quality during the pandemic compared to pre-pandemic, while Sharma et al. [25] revealed less sleep disturbance in preschool children.

Parental Employment and Sleep

Parents’ conditions may influence their children's sleeping patterns. A study conducted by Bernier et al. [26] in 2013 suggested that family socioeconomic status (SES) correlates positively with children’s sleep quality. On the other hand, Top and Cam [27] found no significant association between family income and children’s sleep disturbance during the pandemic. To date, limited data are available evaluating the effect of parents' employment, which represents how much time parents spend at home, on children's sleeping patterns. With more parents spending time at home, it may be hypothesized that more intimate interactions between children and parents occur. However, despite the absence of significant associations between parental employment and sleep duration or quality, the high incidence of sleep disturbance with longer sleep duration may reflect potential issues in psychosocial interaction between parents and children. Various suspected problems include parental stress during work from home, the need to monitor their children’s distance learning needs, maltreatment due to a high workload, and changes in employment status [28]. This indicates that the quality of interaction is more crucial than quantity, warranting further research.

Gadget Exposure, Sleep, and Pandemic

The increased screen exposure, either during the day or before bedtime, has been reported to decrease sleep duration and quality [29]. Studies indicate that screen time in children has increased during the pandemic [30]. Our finding of increased gadget exposure and higher sleep inadequacy and disturbance supported the previous studies. While the increase in gadget exposure before bedtime was linked to sleep disturbance [29], this study did not evaluate whether the increase in sleep disturbance was caused by a shift in learning methods during the pandemic or an increase in pure-recreational screen time. Therefore, further research is needed to distinguish this cause.

Limitations and recommendations 

This study has several limitations. First, the results were self-reported and recalled by the parents of the participants; hence, it was not objectively measured, and biases may appear. Additionally, parents’ physical activity, eating habits, and sleeping patterns, as children's role models, were not assessed in this study. As the results of eating habits during the pandemic between this study and other studies were still conflicting, more data are needed to draw a valid conclusion. Moreover, the data on sleep disturbances before and during the pandemic may need to be collected specifically in future studies. In addition, further exploration of the sleep and wake-up time may reveal the shift in sleeping patterns.

## Conclusions

Altogether, our findings showed that physical distancing during the COVID-19 pandemic affects physical activity, eating habits, and sleeping patterns in preschool children in various ways. During the COVID-19 pandemic, more children were physically inactive. Children experienced changes in eating habits differently based on parental employment, family income and expenses, and the presence of governmental support. While more children had longer sleep duration and more achieved the recommended sleep time, sleep disturbance occurred in most subjects.
